# Prevalence of overweight/obesity and its relationship with metabolic syndrome and fatty liver index in adult patients with type 1 diabetes. A Brazilian multicenter study

**DOI:** 10.1186/s13098-023-00996-0

**Published:** 2023-02-23

**Authors:** Marilia Brito Gomes, Deborah Conte Santos, Karla Drummond, André Pinheiro, Luiza Harcar Muniz, Franz Leal, Carlos Antonio Negrato, Brazilian Type 1 Diabetes Study Group (BrazDiab1SG), Laura Nunes Melo, Roberta Cobas, Lucianne Righeti Monteiro Tannus, Melanie Rodacki, Lenita Zajdenverg, Joana Rodrigues Dantas, Maria Lúcia Cardillo Corrêa-Giannella, Sharon Nina Admoni, Daniele Pereirados Santos, Mariade Fatima Guedes, Sergio Atala Dib, Celso Ferreirde Camargo Sallum Filho, Elisabeth João Pavin, Caroline Takano, Rosângela Roginski Rea, Nicole Balster Romanzini, Mirela Azevedo, Luis Henrique Canani, Hermelinda Cordeiro Pedrosa, Monica Tolentino, Cejana Hamu Aguiar, Reine Marie Chaves Fonseca, Ludmila Chaves Fonseca, Raffaele Kasprowicz, Adriana Costa e Forti, Angela Delmira Nunes Mendes, Renan Montenegro Junior, Virgínia Oliveira Fernandes, João Soares Felício, Flavia Marques Santos

**Affiliations:** 1 Department of Internal Medicine, State University of Rio de Janeiro, Blv. 28 de Setembro, 77, Rio de Janeiro, 20551-030 Brazil; 2 Department of Ophthalmology, Sao Paulo Federal University, Av. Dr. Arnaldo, 455, Cerqueira César, São Paulo, SP Brasil; 3 Department of Ophthalmology, Regional Hospital of Taguatinga. QNC, Área Especial nº 24, Taguatinga Norte/DF, Brasília, Brazil; 4 Department of Ophthalmology, University of Campinas, Rua Tessália Vieira de Camargo, 126, Campinas, São Paulo, Brazil; 5 Medical Doctor Program, University of São Paulo-School of Dentistry, Alameda Dr. Octávio Pinheiro Brisolla, 9-75, Jardim Brasil, Bauru, São Paulo, Brazil

**Keywords:** Type 1 diabetes, Overweight/obesity, Metabolic syndrome, Fatty liver index, Cardiovascular risk factors, Diabetes-related chronic complications

## Abstract

**Aims:**

To determine the prevalence of overweight/obesity and its relationship with metabolic syndrome (MS), fatty liver index (FLI), cardiovascular risk factors (CVRF), and diabetes-related chronic complications (DRCC) in adult patients with type 1 diabetes (T1D).

**Methods:**

This study was conducted in 14 Brazilian public clinics in ten cities, with 1,390 patients: 802 females (57.7%), 779 (56.0%) Caucasians, aged 33.6 ± 10.8 years, age at diagnosis, 16.2 ± 9.2 years, diabetes duration, 17.4 ± 9.2 years, and HbA1c 8.8 ± 2.0%.

**Results:**

Overall, 825 patients (59.4%) had normal weight, and 565 had overweight/obesity; ( 429 (30.9%) presented overweight and 136 (9.8%) presented obesity). After adjustments, overweight/obesity was associated with age, family history of overweight/obesity, total daily insulin dose, hypertension, adherence to diet, type of health care insurance, use of metformin, levels of C-reactive protein, triglycerides, uric acid and HDL-cholesterol. These patients also presented a higher prevalence of MS, FLI ≥ 60, and CVRF than patients without overweight/obesity. Overweight/obesity was not associated with DRCC and with HbA1c levels.

**Conclusions:**

Patients with T1D with overweight/obesity presented traditional risk factors for DRCC, cardiovascular diseases, MS, and non-alcoholic fatty liver disease; most of these risk factors are modifiable and can be avoided with interventions that prevent overweight/obesity.

**Supplementary Information:**

The online version contains supplementary material available at 10.1186/s13098-023-00996-0.

## Introduction

The prevalence of overweight/obesity has more than doubled in many countries since 1980 [1]. This clinical condition is frequently associated with metabolic syndrome (MS) and has been linked to many chronic diseases including type 1 diabetes (T1D) that results from synergistic effects of genetic, immunological, and environmental factors [2]. T1D has been associated with a lean phenotype for a long time [3] but an increasing prevalence of overweight/obesity has been found among these patients in the last decades, with rates varying from 2.8% to 37.1% [4–6]. These differences seem to occur due to different definitions of obesity, age range, and ethnicity among the studies [7]. Different types of insulin therapeutic regimens (ITRs) have also been associated with overweight/obesity, as observed in the Diabetes Control and Complications Trial (DCCT) where the use of intensive insulin therapy was closely associated with weight gain [8, 9].

In general, patients with overweight/obesity show other components of insulin resistance or MS that are associated with poor glycemic control [10], diabetes-related chronic complications (DRCC) and non-alcoholic fatty liver disease (NAFLD) [11–13]. A recent meta-analysis found that approximately one quarter (23.7%) of patients with T1D were affected by MS [14] and almost 22% had NAFLD [15]. The presence of MS or insulin resistance are considered risk factors for chronic kidney disease (CKD), cardiovascular disease [11], and diabetic retinopathy (DR) [12]. Obese patients with MS present a high risk of developing NAFLD, one of the most important causes of liver damage [16]. Recently, a study carried out in a tertiary care center in our country [13] has shown that patients with T1D and altered hepatic images on ultrasound or transient elastography, had higher body mass index (BMI) and higher prevalence of MS [13]. However, both tests are not easy to perform in routine clinical practice and some markers of liver impairment such as high levels of transaminases and gamma-glutamyl-transferase (GGT) lead to the assumption of NAFLD in individuals that present MS [13, 17]. Nevertheless, in many patients with MS, these biomarkers may be found within the normal range [13, 17]. Recently the fatty liver index (FLI) was developed and validated in an Italian population [18] and became a useful tool helping physicians select those patients who should undergo liver ultrasonography.

The aim of this study was to investigate the prevalence of overweight and/or obesity and its relationship with MS, FLI, cardiovascular risk factors (CVRF), and DRCC in Brazilian adult patients with T1D.

## Subjects, materials and methods

This was a cross-sectional study conducted in 14 public clinics from several regions of Brazil between 2011/2014.

All patients received health care from Brazilian National Health Care System (BNHCS). Each clinic provided data from at least 50 T1D outpatients that were treated by an endocrinologist in secondary or tertiary care settings. Included patients were those with the diagnosis of T1D done by a physician and needing continuous insulin use since diagnosis, aged ≥ 10 years, and followed at each diabetes center for at least 6 months. Pregnant or lactating women, patients who had an acute infection or ketoacidosis in the three preceding months or had a history of renal transplantation were excluded [19].

The total sample consisted of 1390 adult patients. Each center had a local ethics committee that approved the study. Patients and/or their parents where necessary, signed a written informed consent agreeing with the participation in the study.

The collected data (demographic, clinical and laboratory) are described in Table 2. Self -reported color-race (White, Black, Brown (“parda”), Asian (“amarela”) and Indigenous (“indígena”)) [20], type of prescribed insulin therapeutic regimens (ITRs), self-reported adherence to diet (following at least 80% of the time the prescribed diet) [19, 21] were also evaluated.

Adequate glycemic control was defined as HbA1c levels < 7.0% (58 mmol/mol) [22]. HbA1c was measured using high-performance liquid chromatography (Bio-Rad Laboratories, Hercules, California, USA). The last value of HbA1c in the previous year was obtained from medical records. Fasting triglycerides, HDL cholesterol, total cholesterol, alanine aminotransferase (ALT), aspartate aminotransferase (AST) and GGT were measured using enzymatic techniques and serum uric acid by a uricase-based commercial kit (mg/dL). For ALT and AST, we considered normal values < 25 U/L for women and < 33 U/L for men [23]. Creatinine was measured using a colorimetric assay kit, corrected for a standardized creatinine assay by mass spectrometry. C-reactive protein (CRP) was measured using a high sensitive enzyme immune-assay and expressed in mg/dL (BioSystem, model A25; Barcelona, Spain). Friedewald’s equation was used to calculate LDL cholesterol [24]. ITRs were stratified as follows: exclusive use of intermediate insulin (NPH) or regular insulin, long-acting insulin analogs plus short-acting insulin, or continuous subcutaneous insulin infusion (CSII). BMI was classified as underweight (< 18.5 kgm^2^), normal (≥ 18.5 to < 25 kgm^2^), overweight (≥ 25 to < 30 kgm^2^) and obese (≥ 30 kgm^2^) according to the World Health Organization criteria [25]. Hypertension was defined as sustained blood pressure ≥ 140/90 mmHg or the current use of antihypertensive drugs [22]. Current smoking was defined as the use of more than one cigarette per day.

The diagnosis of MS was done according to the International Diabetes Federation criteria [26]. Considering that all participants had diabetes, central obesity (waist circumference ≥ 90 cm in South American men or ≥ 80 cm in South American women) plus an additional factor was necessary for diagnosing MS (Triglycerides ≥ 150 mg/dL (1.7 mmol/L) or on drug therapy for elevated triglycerides; HDL < 40 mg/dL (1.03 mmol/L) in men or < 50 mg/dL (1.29 mmol/L) in women or on drug therapy for low HDL; elevated BP (systolic or diastolic) ≥ 130/85 mmHg or the use of antihypertensive drugs) [26].

The risk of presenting fatty liver was determined by the FLI [18]. Participants with FLI ≥ 60 and < 30 were classified as having high and low risk of having a fatty liver, respectively. Values between 30 and 60 were considered as undetermined risk [18].

Sample calculation has been previously described [19, 27]. It represents the distribution of T1D cases across four geographic regions of Brazil, estimated using the overall population distribution reported in the 2010 Brazilian Institute of Geography and Statistics Population Census (IBGE) [28], combined with national estimates of diabetes prevalence, to determine the minimum number of patients to be studied in each region [29]. Economic status was defined according to the Brazilian Economic Classification Criteria that takes in account the education level [30]. The following economic status were considered: high, middle, low, and very low.

Renal function was estimated by the CKD-EPI equation [31] in patients with age ≥ 16 years and was expressed as estimated glomerular filtration rate (eGFR) in milliliters per minute per 1.73m^2^ (mL/min/1.73m^2^). Albuminuria concentration (immunoturbidimetry, detection limit:0.01 mg/dL) was measured at least twice in a morning urine sample. The presence of albuminuria was defined as a value ≥ 30 mg/dL. Patients with normal renal function had an eGFR ≥ 60 mL/min/1.73m^2^ and the absence of albuminuria. CKD was defined as an eGFR < 60 mL/min/1.73m^2^, with or without the presence of albuminuria and an eGFR ≥ 60 mL/min/1.73m^2^ with the presence of albuminuria [32].

Screening for DR was performed by mydriatic binocular indirect ophthalmoscopy [BIO; EyeTec (OBI OSF)]. The classification of DR was assessed in the eye that was the most compromised. Each eye was classified based on whether DR was present. Patients were then classified according to the international classification as absent, non-proliferative diabetic retinopathy (NPDR), proliferative diabetic retinopathy (PDR) and macular edema [33].

For statistical analysis purposes, overweight and obesity were grouped. An exploratory analysis was initially performed, and the data were presented as mean (± SD) or median, interquartile range [IQR] for continuous variables and percentage for discrete variables. Parametric and non-parametric tests were used for comparison between the groups as indicated. Pearson’s correlation coefficient was calculated when applicable.

We performed backward Wald logistic multivariate analysis to explore the variables associated with overweight/obesity considered as a dependent variable (outcome variable); for the first model, we have used independent variables, with p < 0.2 in exploratory analysis mainly related to demographic and social data, or those which presented relevance, such as gender, age, diabetes duration, time of follow-up at each center, years of school attendance, self-reported color-race, family history of obesity, of type 2 diabetes (T2D) and hypertension, total daily insulin dose (U/day), basal/bolus ratio, type of health care insurance and adherence to diet. Interaction between family history of obesity, T2D, and hypertension was also evaluated.

In the second model, we have used independent variables, with p < 0.2 in exploratory analysis mainly related to clinical and laboratory data such as age, diabetes duration, presence of hypertension, use of antihypertensive drugs, statins, metformin and levels of triglycerides, HDL cholesterol, total cholesterol, ALT, AST, GGT, CRP and uric acid. The first and second models were adjusted for gender, age at diabetes diagnosis, social-economic status, self-reported color-race, and geographic region of the country.

All analyses were performed using the Statistical Package for the Social Sciences (SPSS version 17.0, SPSS, Inc., Chicago, Illinois, USA). Odds ratios with 95% confidence intervals (CIs) were calculated where indicated. A two-sided *p-value* less than 0.05 was considered significant.

## Results

Overall, 825 patients (59.4%) had normal weight, and 565 had overweight/obesity;[ 429 (30.9%) presented overweight and 136 (9.8%) presented obesity]. No patient had underweight. FLI was low in 892 (64.2%), undetermined in 255 (18.3%) and high in 163 (11.7%) patients. The sociodemographic data of the studied population are listed in Additional file 1: Table S1.

Clinical, demographic, and laboratory data stratified according to the presence of overweight/obesity are described in Table 1. Overall, patients with overweight/obesity, were older, had longer diabetes duration, higher age at diabetes diagnosis, had a long time of follow-up in the respective center, belonged more frequently to medium socioeconomic status, and were less adherent to the prescribed diets. Clinically, they had higher BMI, larger waist circumference, had more frequently acanthosis nigricans and MS, as well as higher rates of hypertension, higher levels of sBP, dBP, uric acid, total cholesterol, triglycerides, CRP, LDL cholesterol, creatinine, AST and ALT, FLI and FLI ≥ 60. They also showed lower levels of HDL cholesterol and eGFR. These patients used higher daily insulin doses, were more frequently using metformin, anti-hypertensive drugs, and statin, had more frequently a family history of overweight/obesity, T2D, hypertension, and coronary disease without difference in the prevalence of DR and CKD. Overall, 73 patients (5.4%) presented altered levels of AST or ALT, which was found to be higher in patients with overweight/obesity compared to patients with normal weight, 39 (7.1%) vs 34 (4.2%), p = 0.02, respectively, and in women compared to men 50 (68.5%) vs 23 (31.5%), p = 0.047, respectively.

**Table 1:** Clinical, demographic and laboratory data stratified by the presence of overweight/obesity

	Overweight/obesity		
	No	Yes	*p-value
N (%)	825 (59.4)	565 (40.6)	
Demographic data			
Gender, female n (%)	470(57.0)	332 (58.8.0)	0.5
Age, year	32.7 ± 11.03	34.9 ± 10.4	< 0.001
Diabetes duration, year	16.88 ± 9.2	18.3 ± 9.3	0.002
Age at diagnosis, year	16.1 ± 9.0	16.5 ± 9.4	0.03
Time of follow up, year	8.6 [9.2]	11.3. [10.9]	< 0.001
Level of care, tertiary n (%)	531(64.4)	375 (66.4)	0.4
Health care insurance(public and private), yes n(%)	238 (28.8)	191(33.8)	0.05
Years of study, year	10.9 ± 2.5	10.8 ± 2.3	0.7
Smoker, yes n(%)	45(5.5)	30(5.3)	0.05
Ethnicity, y (%)^a^			
Caucasian	465 (56.4)	314 (55.6)	0.7
Geographic region, n (%)			0.3
Southeast	395 (47.9)	277(49.9)	
South	121(14.7)	89(15.8)	
North/Northeast	225(27.3)	131(23.2)	
Mid-west	84(10.2)	68(12.2)	
Economic status (%)			0.03
High	18(2.2)	27 (4.8)	
Medium	378(45.8)	271(48.0)	
Low	402(48.7)	252(44.6)	
Very Low	27(3.3)	15(2.7)	
Diabetes management and treatment			
HbA1c (%)	8.8 ± 2.1	8.8 ± 1.8	0.5
HbA1c (mmol/mol)	73.3 ± 23.1	72.7 ± 19.8	
HbA1c < 7.0% n (%)	129(28.8)	73(24.8)	0.4
HbA1c (%) year before HbA1c (mmol/mol), year before	8.8 ± 2.2 72.9 ± 24.0	8.9 ± 1.8 71.8 ± 20.4	0.4
Insulin dose (U//day)	50,1 ± 22.4	60.7 ± 25.1	< 0.001
Insulin dose (U/kg/day)	0.84 ± 0.4	0.80 ± 0.31	0.05
SMBG, yes n (%)	776(94.1)	527 (93.3)	0.7
SMBG, n	3.6 ± 1.5	3.7 ± 1.5	0.4
Adherence to diet, yes n(%)	465 (63.3)	275(56.6)	0.02
Physical activity, yes n(%)	393(47.7)	280(49.6)	0.4
Number of clinical visits/year	3.7 ± 1.7	3.6 ± 1.7	0.7
Insulin Therapeutic regimen (ITR), n(%)			0.2
NPH or NPH + regular	744 (90.2)	520(92.0)	
Insulin analogs (long or short-acting)	46(5.6)	31(5.5)	
CSII	35(4.2)	14(2.5)	
Bolus (%)	0.32 ± 0.15	0.33 ± 0.14	0.4
Adherence to ITR, yes n(%)	95(19.9)	53(16.9)	0.3
Clinical data			
BMI, kg/m^2^	22,2 ± 2.2	28,3 ± 3.5	< 0.001
Waist circumference(WC), cm	78.4 ± 8.3	92.6 ± 10.5	< 0.001
Systolic blood pressure	122.5 ± 16.3	127.1 ± 15.9	< 0.001
Diastolic blood pressure	75.5 ± 10.3	78.1 ± 9.8	< 0.001
Hypertension, yes n(%)	144 (17.5)	165 (29.3)	< 0.001
Metabolic Syndrome data			
Metabolic syndrome, yes n(%)^c^	119(15.1)	323 (60.5)	< 0.001
Waist circumference, yes n (%)	170 (21.1)	428(77.8)	< 0.001
Acanthosis yes n(%)	15(1.9)	48(8.7)	< 0.001
Blood pressure ≥ 130/85 mmHg, yes n(%)	313 (37.9)	303(53.6)	< 0.001
Triglycerides (mg/dL)	80[51.0]	108 [96.5]	< 0.001
High triglycerides, yes n(%)	99(12.2)	131(23.9)	< 0.001
HDL-cholesterol (mg/dL)	58.3 ± 17.7	53.9 ± 21.0	0.008
Low HDL-Cholesterol, yes n(%)	183 (22.6)	173(31.6)	< 0.001
Laboratory data			
Uric acid (mg/dL)	4.9 ± 1.7	5.5 ± 2.2	< 0.001
Total Cholesterol (mg/dL)	185.3 ± 44.9	193.9 ± 56.0	0.002
LDL-cholesterol (mg/dL)	108.1 ± 39.1	113.2 ± 42.2	0.02
LDL-cholesterol < 100 mg/dl),yes n(%)	447(55.4)	313(56.6)	0.7
Non-HDL-cholesterol(mg/dL)	127.1 ± 43.6	138.7 ± 49.1	< 0.001
ALT, U/L	11.0 [7.0]	13.0 [10.0]	< 0.001
AST, U/L	17.0 [9.0]	19.0[12.0]	0.001
GGT, mg/dL	18.0[14.0]	21.0[17.0]	0.5
Creatinine (mg/dL)	1.04 [0.33]	1.2 [0.35]	0.001
C reactive protein (mg/dL)	0.13 [0.31]	0.32[0.69]	0.001
Medications			
Metformin, yes n(%)	54 6.5)	124(21.9)	< 0.001
Anti-hypertensive drugs, yes n(%)	224(27.4)	220(39.2)	< 0.001
Statin yes n(%)	183(22.2)	179(31.7)	< 0.001
Family history			
Overweight/obesity, yes n(%)	158(19.2)	174(30.8)	< 0.001
Diabetes type 2, yes n(%)	224(27.2)	181(32.0)	0.049
Hypertension	472(57.2)	358(63.4)	0.02
Coronary disease	138(16.7)	126(22.3)	0.009
Diabetes-related chronic complications			
Retinopathy, yes n (%)	320(41.9)	240 (45.1)	0.2
FLI	9.9[12.5]	39.4[43.4]	< 0.001
FLI, ≥ 60,n (%)	11(1.4)	152(28.8)	< 0.001
CKD, yes n (%)	222(27.2)	166(29.7)	0.3
GFR, mL/min/1.73m^2b^	81.3 ± 26.0	78.3 ± 25.2	0.03
Albuminuria, mg/dL	9.2[22.8]	9.4[22.7]	0.4

The data are presented as n (%), mean ± SD or median [IQR, interquartile range];* p < 0.05 was considered significant

*ALT* alanine aminotransferase, *AST* aspartate aminotransferase, *GGT* gamma-glutamyl transferase, *FLI* fatty liver index

^a^African-Brazilians, Mulattos, Asians, Native Amerindians were considered as non-Caucasians.,^**††**^Physical activity, at least 3/ times per week.

^b^Glomerular filtration rate

^c^Metabolic syndrome was defined according to International Diabetes Federation (WC ≥ 90 cm in South American men or ≥ 80 cm in South American women; triglycerides ≥ 150 mg/dL (1.7 mmol/L) or on drug therapy for elevated triglycerides; HDL < 40 mg/dL (1.03 mmol/L) in men or < 50 mg/dL (1.29 mmol/L) in women or on drug therapy for low HDL; elevated blood pressure (systolic or diastolic) ≥ 130/85 mmHg or the use of antihypertensive drugs)

Considering the group of patients with all data available, regarding the components of MS (n = 1,323), 670 (48.2%) did not present overweight/obesity or MS, and 323 (23.2%) had both clinical conditions. MS without overweight/obesity was observed in 119 (9%) and overweight/obesity without the presence of MS in 211 (15.2%) patients. MS was found in 442 patients (31.8%) being high blood pressure its most prevalent component (44.3%), followed by low HDL-cholesterol (26.2%) and high triglycerides levels (16.9%). A higher number of components of MS was noted in patients with overweight/obesity compared to patients with normal weight, one [1] vs two [1], p < 0.001, respectively. In the whole sample, 175 (13.4%) patients presented three or more components of MS.

**Fig. 1:**
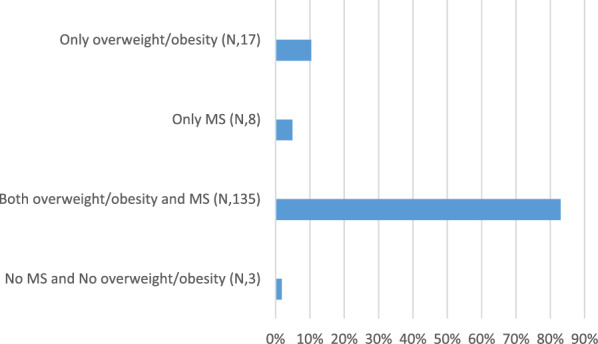
Pevalence of MS and overweight/obesity in the subgroup of patients with FLI ≥ 60 (N = 163)

Figure 1 shows the prevalence of MS and overweight/obesity in the subgroup of patients with FLI ≥ 60 (N = 163). A higher prevalence of FLI ≥ 60 was observed in patients with both overweight/obesity and MS, 135 (82.8%), compared to patients without both conditions, 3 (1.8%), to patients with MS without overweight/obesity, 8 (4.9%), and to patients with overweight/obesity without MS, 17 (10.4%), p < 0.001.

No difference was noted concerning the level of HbA1c in the year the evaluation was conducted as well as in the previous year. A correlation was noted between the last values of HbA1c in the previous year with HbA1c values measured during the study (r = 0.84, p < 0.001) among the 1,200 patients that had both measures. HbA1c values obtained during the study were correlated with the levels of total cholesterol (r = 0.163, p < 0.001), triglycerides (r = 0.140, p < 0.001), ALT (r = 0.099, p = 0.001), AST (r = 0.109, p = 0.001), GGT ((r = 0.159, p < 0.001), LDL-C (r = 0.077, p = 0.005), FLI (r = 0.057, p = 0.040) and waist circumference (r = − 0.073, p = 0.007). No correlation was found between HDL-C values and HbA1c.

The first model of multivariate analysis performed with the presence of overweight/obesity as a dependent variable showed that all the demographic and social independent variables which entered in the model could explain 10.4% (Nagelkerke R-squared) of a given patient having overweight/obesity. 61.5% of the patients were correctly classified by the model. The presence of overweight/obesity was associated with age, adherence to diet, family history of obesity, and type of health care insurance (Table 2).

**Table 2:** Final model of the first model of logistic regression with overweight/obesity as a dependent variable and social and demographic data as independent variables

Variable	B	OR	95% confidence interval	p value
Age, years	0.017	1.019	1.006–1.029	0.01
Adherence to diet, yes	− 0.277	0.758	0.587–0.977	0.03
Private and public health care insurance, yes	− 0.301	0.740	0.568–0.964	0.02
Familiar history of obesity, yes	0.569	1.766	1.328–2.348	0.001
Insulin dose (U//daily)	0.020	1.020	1.014–1.025	0.001

The second model performed with the presence of overweight/obesity as a dependent variable showed that all clinical and laboratory independent variables which entered in the model could explain 14.0% (Nagelkerke R-squared) of a given patient having overweight/obesity. 66.5% of the patients were correctly classified by the model. The presence of overweight/obesity was associated with age, hypertension, use of metformin, lower levels of HDL-Cholesterol, higher levels of uric acid, triglycerides, and C-reactive protein (Table 3).

**Table 3:** Final model of the second model of logistic regression with overweight/obesity as a dependent variable and clinical and laboratory data and use of medications as independent variables

Variable	B	OR	95% confidence interval	p value
Age, years	0.015	1.015	1.003–1.027	0.015
Presence of hypertension, yes	0.413	0.758	1.110–2.059	0.009
Use of metformin,yes	1.252	3.496	2.435–5.018	0.001
HDL-cholesterol (mg/dL)	− 0.009	0.991	0.984–0.998	0.015
Triglycerides (mg/dL) Uric acid (mg/dL	0.003 0.113	1.003 1.119	1.001–1.005 1.038–1.208	0.011 0.004
C-reactive Protein	0.101	1.106	1.012–1.209	0.027

First and second model were adjusted for social-economic status, self-reported color-race and age at diabetes diagnosis, and gender.

## Discussion

The present study showed that approximately 40% of adult patients with T1D presented overweight/obesity. These patients had a higher prevalence of hypertension, dyslipidemia, MS, increased levels of uric acid, inflammatory biomarkers, and FLI ≥ 60 that are considered traditional risk factors for DRCC, cardiovascular diseases, and NAFLD. The presence of overweight/obesity was also associated with lower adherence to diet, increased use of metformin, family history of obesity in first-degree relatives, use of higher total daily insulin dose, and type of health care insurance.

Although no association was found with glycemic control (current and in the previous year) it is important to emphasize that less than 30% of the patients in both groups presented an adequate glycemic control. So, most patients were adding another important CVRF (inadequate glycemic control) to the already preexisting risk factors. No association was found between overweight/obesity with some DRCC such as CKD and DR. The above-mentioned data show that these patients had higher prevalence of many risk factors associated with micro and macrovascular DRCC than patients with normal weight.

Overweight/obesity affects many adult patients with T1D across their lifetime (37 to approximately 80%) [4], with a prevalence that varies according to the country, gender, and age range. Welters et al., have also found an increase in the mean BMI in a German/Austrian adult population with T1D, between 1999 and 2018, mostly pronounced among patients aged 21–55 years, with higher baseline BMI [34]. They have also found that the prevalence of obesity is increasing faster in this population compared to the general German background population. Similar data were observed in the Pittsburgh Epidemiology of Diabetes Complications (EDC) Study [35]. However, both studies were longitudinal and not cross-sectional as the present one. Although the prevalence of overweight/obesity found in our study among patients with T1D was pretty high (approximately 40%), it was lower than the values found in the general population in the Brazilian Longitudinal Study of Adult Health (ELSA-Brasil), a multicentric cohort study, that has found that 63.1% of the participants had excess body weight [36].

No relationship between self-reported color-race and obesity/overweight was found in this study unlike data found in a study conducted in the USA with a large sample of patients with T1D in which obesity rates were higher among African-Americans [37]. However, it is important to highlight that patients who self-reported as Black in this study, presented greater heterogeneity regarding genomic ancestry (less African and more European genomic ancestry) than that found among African-Americans [38].

Our data did not show an association between overweight/obesity with HbA1c levels or with the number of patients that reached the targets for good glycemic control, similar to data found in another study [39], in which patients with overweight/obesity used higher daily insulin doses compared to patients without overweight/obesity, but also with no difference in HbA1c levels.

Controversial results have been described in patients with T1D with overweight/obesity concerning glycemic control [8, 39]. The DCCT showed that independent of the weight gain, patients under intensive insulin treatment achieved the same HbA1c levels during the follow-up [8]. However, the above mentioned studies that have focused on the levels of HbA1c in patients with T1D, with and without overweight/obesity, have found a difference in HbA1c levels no greater than 0.5%.

Although most of our patients were under the use of multiple insulin injections, less than 20% reported adherence to IRTs, and less than 50% reported adherence to diet which could have an impact upon glycemic control. Overall, patients with overweight/obesity were less adherent to the prescribed diet as has been described in FinnDiane Study, also conducted with adult patients with T1D [38]. Several factors permeate the adherence to the prescribed diet, such as socioeconomic factors, perceived stress, knowledge about the disease, and even the type of health care insurance [21, 27, 40, 41].

Regarding the type of health care insurance, in the present study, patients who had public and private health care insurance had higher prevalence of overweight and obesity possibly because these patients generally belong to higher socioeconomic status, have more access to food supply, and consequently higher caloric intake.

MS was present in 31.8% of the patients, with higher prevalence in the group with overweight/obesity. These patients have a prevalence of MS that is in the range observed in the general Brazilian population which varies from 8.9% to 38.1% [42], as well as in the range found in a recent meta-analysis including only patients with T1D [14], in which a large heterogeneity in the prevalence of MS was found, varying from 3.2% (Poland) to 57.1% (Finland) [14]. It is noteworthy that in the present sample,15.2% of the patients with obesity did not present MS.

Patients with overweight/obesity had higher prevalence of the components of MS in comparison to patients without this condition. The most prevalent component of MS was elevated blood pressure, followed by low HDL-cholesterol and high triglycerides, which is one of the most prevalent findings in the general Brazilian population [42], as well as in patients with T1D that participated in the FinnDiane study [10]. In our sample, 175 (13.4%) patients presented three or more components of MS like data observed in FinnDiane study [10]. The role of elevated blood pressure in the development of micro and macrovascular complications in patients with T1D is well known and has been observed in many studies [10, 11].

Like other studies performed with patients presenting T1D, those with overweight/obesity presented higher levels of total and LDL-cholesterol and triglycerides, which result in a more atherogenic lipid profile and thus higher risk for cardiovascular disease [43]. The cardiovascular risk can be even higher when considering FLI that has been validated only in the general Italian population [18], but it was used as a surrogate marker of NAFLD in Spanish patients with T1D [44]. In this latter study, an association between this index with lipid profile was found, especially with VLDL lipoprotein. In a recent study conducted in our country, higher FLI was found in patients with T1D with altered hepatic image either by ultrasound or transient elastography [13]. In the present study, patients with overweight/obesity and MS had a higher prevalence of FLI ≥ 60 showing an increased risk of having NAFLD.

Finally, the use of metformin as an adjunct therapy to insulin was higher in patients with overweight/obesity, mainly females (data not shown). A study showed that metformin use was associated with significant reductions in HbA1c levels and insulin doses, with no significant changes in weight [45]. Another study, conducted in Denmark, did not find a significant difference in HbA1c levels, but the insulin doses and weight showed significant reductions with metformin use [46]. The difference found between the results could be attributed to the studies’ design which was longitudinal for the formers and cross-sectional for the present one.

As expected, patients with overweight/obesity were more frequently using anti-hypertensive drugs and statins due to their associated comorbidities.

The strength of this study was mostly due to the fact that a large cohort of ethnically admixed patients from different geographic regions of Brazil was uniformly evaluated and had their data analyzed. To the best of our knowledge, this is the largest study ever conducted in Brazil regarding obesity/overweight in adult patients with T1D.

However, our data should be considered with some caution due to the cross-sectional nature of the study that does not allow us to ascertain causation. We have also used only clinical criteria for the diagnosis of T1D and we did not perform measurement of C peptide and autoantibodies against beta cells similar to other epidemiological studies [12, 43, 47]. The information about adherence to diet and ITRs was self-reported which could lead to some bias. Finally, we did not have the basal BMI of these patients, so we are not able to determine their BMI evolution.

In conclusion, almost 40% of our patients, presented overweight/obesity that was associated with higher prevalence of some traditional risk factors for DRCC and cardiovascular diseases. Besides these alterations, these patients also presented high levels of inflammatory biomarkers, high prevalence of MS and FLI ≥ 60 which are risk factors for the presence of NAFLD. Considering that these risk factors have a great impact on morbidity and mortality rates and are modifiable, diabetes teams should rethink their approach to these patients, offering interventions that prevent overweight/obesity.

 ≠ Brazilian Type 1 Diabetes Study Group (BrazDiab1SG) participants are described in Additional file 1: Table S2.

## Supplementary Information

**Additional file 1: Table S1.** Clinical and demographic data of the studied population. **Table S2.** Brazilian Type 1 Diabetes Study Group (BrazDiab1SG).

## Data Availability

The used datasets and/or analyzed during the current study are available with the corresponding author upon reasonable request.
