# Supplementary material for: Prevalence of overweight/obesity and its relationship with metabolic syndrome and fatty liver index in adult patients with type 1 diabetes. A Brazilian multicenter study
Source: Diabetol Metab Syndr. 2023 Feb 23;15:28. doi: 10.1186/s13098-023-00996-0 (PMC9948365; doi:10.1186/s13098-023-00996-0)
Supplement: Supplementary file 1 — Additional file 1: Table S1. Clinical and demographic data of the studied population. Table S2. Brazilian Type 1 Diabetes Study Group (BrazDiab1SG). [file 13098_2023_996_MOESM1_ESM.docx]

**Supplementary table 1.** Clinical and demographic data of the studied population.

| **Variable** |  |
| --- | --- |
| **N** | 1,390 |
| **Age, y** | 33.6 ± 10.8 |
| **Gender, female, n (%)** | 802 (57.7) |
| **Age at diabetes diagnosis, y** | 16.2 ± 9.2 |
| **Years of study, y** | 12.5 ± 3.9 |
| **Diabetes duration, y** | 17.4 ± 9.2 |
| **HbA1c (%)** | 8.8 ± 2.0 |
| **Ethnicity, n (%)** |  |
| Caucasian | 779 (56.0) |
| **Geographic region, n (%)** |  |
| Southeast | 672 (48.3) |
| North/northeast | 356 (25.6) |
| South | 210 (15.1) |
| Mid-west | 152 (10.9) |
| **Economic status** |  |
| High | 45 (3.2) |
| Medium | 649 (46.7) |
| Low | 654 (47.1) |
| Very low | 42 (3.0) |
| **Level of care n (%)** |  |
| Secondary | 484 (34.8) |
| Tertiary | 906 (65.2) |
| **Time of follow-up, y** | 9.2[10.0] |
| **Health insurance (%)** |  |
| Public only | 961 (69.1) |
| Public and private | 429 (30.9) |

y = year; data are presented as number (percentage), mean ± SD or media [interquartile range ,IQR]

* African-Brazilians, Mulattos, Asians, and Native Indians.

#Supplementary table 2 : Brazilian Type 1 Diabetes Study Group (BrazDiab1SG)

Executive steering committee: Marilia Brito Gomes (chair), Carlos Antonio Negrato.

Principal investigators are indicated by an asterisk.

|  |  |  |
| --- | --- | --- |
| Marilia Brito Gomes* | State University of Rio de Janeiro | mariliabgomes@gmail.com |
|  |  |  |
| Laura Nunes Melo | State University of Rio de Janeiro | lauragnmelo@gmail.com |
| Roberta Cobas | State University of Rio de Janeiro | robertacobas@gmail.com |
| Lucianne Righeti Monteiro Tannus | State University of Rio de Janeiro | luciannetannus@ig.com.br |
| Melanie Rodacki* | Federal University Hospital of Rio de Janeiro | mrodacki2001@yahoo.com.br |
| Lenita Zajdenverg | Federal University Hospital of Rio de Janeiro | lenitazaj@gmail.com |
| Joana Rodrigues Dantas | Federal University Hospital of Rio de Janeiro | joanardantasp@ig.com.br |
| Maria Lúcia Cardillo Corrêa-Giannella* | University Hospital of São Paulo | malugia@lim25fm.usp.br |
| Sharon Nina Admoni | University Hospital of São Paulo | sharonadmoni@ gmail.com |
| Daniele Pereira dos Santos | University Hospital of São Paulo | dps.daniele@ hotmail.com |
| Carlos Antonio Negrato* | Bauru’s Diabetics Association | carlosnegrato@uol.com.br |
| Maria de Fatima Guedes | Bauru’s Diabetics Association | tatiguedeses@hotmail.com |
| Sergio Atala Dib* | Federal University of São Paulo State | sergio.dib@unifesp.br |
| Celso Ferreira de Camargo Sallum Filho | Federal University of São Paulo State | celsosallum@superig.com.br |
| Elisabeth João Pavin* | University of Campinas | [ejpavin@fcm.unicamp.br](mailto:ejpavin@fcm.unicamp.br) |
| Caroline Takano | University of Campinas | caroline.takano@gmail.com |
| Rosângela Roginski Rea* | Federal University of Paraná | rosangelarea@uol.com.br |
| Nicole Balster Romanzini | Federal University of Paraná | nikbr@hotmail.com |
| Mirela Azevedo* | Clinical Hospital of Porto Alegre | mirelajobimazevedo@gmail.com |
| Luis Henrique Canani | Clinical Hospital of Porto Alegre | luishenriquecanani@gmail.com |
| Hermelinda Cordeiro Pedrosa* | Regional Hospital of Taguatinga | pedrosa.hc@globo.com |
| Monica Tolentino | Regional Hospital of Taguatinga | monicatolentino@uol.com.br |
| Cejana Hamu Aguiar | Regional Hospital of Taguatinga |  |
| Reine Marie Chaves Fonseca* | Diabetes and Endocrinology Center of Bahia | reinemar@terra.com.br |
| Ludmila Chaves Fonseca | Diabetes and Endocrinology Center of Bahia |  |
| Raffaele Kasprowicz | Diabetes and Endocrinology Center of Bahia | raffaellebarros@hotmail.com |
| Adriana Costa e Forti* | Diabetes and Hypertension Center of Ceará | adrianaforti@uol.com.br |
| Angela Delmira Nunes Mendes | Diabetes and Hypertension Center of Ceará | (angeladelmira@terra.com.br) |
| Renan Montenegro Junior* | Federal University of Ceará | renanjr@ufc.br |
| Virgínia Oliveira Fernandes | Federal University of Ceará | (virginiafernande@hotmail.com |
| João Soares Felício* | Federal University Hospital of Pará | felicio.bel@terra.com.br |
| Flavia Marques Santos | Federal University Hospital of Pará | [drafms@bol.com.br](mailto:drafms@bol.com.br) |
